# Pycnogenol Ameliorates Depression-Like Behavior in Repeated Corticosterone-Induced Depression Mice Model

**DOI:** 10.1155/2014/942927

**Published:** 2014-05-13

**Authors:** Lin Mei, Miyako Mochizuki, Noboru Hasegawa

**Affiliations:** ^1^School of Food and Biological Engineering, Jiangsu University, Zhenjiang 212013, China; ^2^Kyoto Bunkyo Junior College, 80 Senzoku, Makishima-cho, Uji, Kyoto 611-0041, Japan; ^3^Department of Health and Medical Sciences, Ishikawa Prefectural Nursing University, 1-1 Gakuendai, Kahoku, Ishikawa 929-1210, Japan

## Abstract

Oxidative stress is considered to be a mechanism of major depression. Pycnogenol (PYC) is a natural plant extract from the bark of *Pinus pinaster* Aiton and has potent antioxidant activities. We studied the ameliorative effect of PYC on depression-like behavior in chronic corticosterone- (CORT-) treated mice for 20 days. After the end of the CORT treatment period, PYC (0.2 mg/mL) was orally administered in normal drinking water. Depression-like behavior was investigated by the forced swimming test. Immobility time was significantly longer by CORT exposure. When the CORT-treated mice were supplemented with PYC, immobility time was significantly shortened. Our results indicate that orally administered PYC may serve to reduce CORT-induced stress by radical scavenging activity.

## 1. Introduction

Stress is a potent risk factor for depression. Recent studies have reported that plasma reactive oxygen species (ROS) content increased in major depression patients [1], and oxidative stress is contributive to major depression [2]. Chronic high levels of glucocorticoids lead to maladaptive anxiety depressive disorders [3]. Stress-based animal models by chronic corticosterone (CORT) treatment might mimic stress-triggered depression in humans [4], and accumulation of oxidative stress has been noted in the brain of stress-induced animal models [5]. A forced swimming test (FST) was developed as an animal model of depression to investigate the effects of new antidepressant compounds in mice [6]. FST has very good reproducibility and is very commonly used by researchers [6].

Pycnogenol (PYC), a family of flavonoids isolated from French maritime pine bark (*Pinus pinaster* Aiton, synonym* Pinus maritima* Mill.), is a mixture of procyanidins with potent antioxidants and ROS scavenging properties [7]. As yet there has been no report about the antidepressant effect of PYC. Thus, the present study was conducted to evaluate the efficacy of PYC in protecting against chronic depression in CORT-induced depression mice model using FST.

## 2. Materials and Methods

### 2.1. Animals and Chemicals

**Figure 1:**
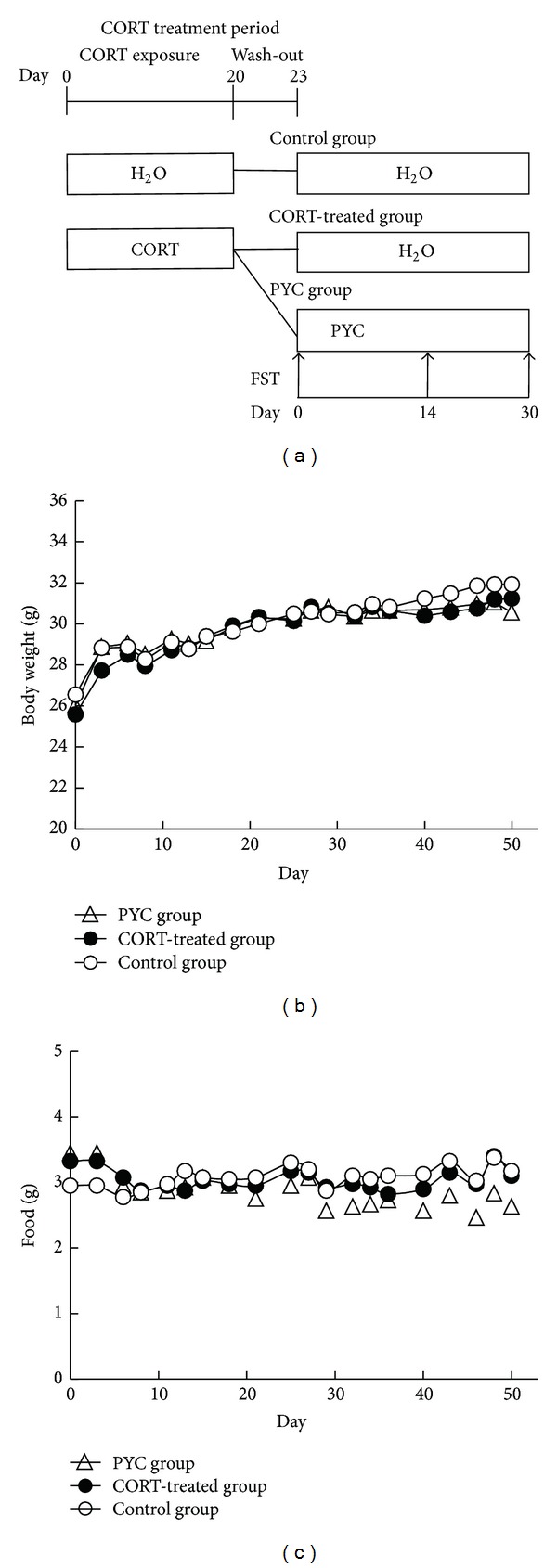
Effect of PYC and CORT on body weight and food intake. (a) Timetable: CORT was administered from 0 to 20 days. PYC was administered from 23 to 30 days. (b) Body weights. (c) Food intake. Results are presented as the mean of six experiments.

Eighteen male C57Bl/6J mice weighing 25.3 ± 0.71 g were obtained from Charles River Japan Inc. (Tokyo, Japan). The mice were housed in standard cages and fed a commercial diet (Oriental Yeast Co., Ltd., Tokyo, Japan) and tap water ad libitum. The depression model was induced by repeated administration of CORT (14 days of 6.9 mg/kg, 3 days of 3.1 mg/kg, and then 3 days with 1.6 mg/kg) in the drinking water over a period of 20 days according to the method of Gourley et al. [8]. The control group (*n* = 6) received normal drinking water. Three days after CORT, the mice were randomly separated into 2 groups: the PYC group (*n* = 6) received PYC at 0.2 mg/mL; the CORT-treated group (*n* = 6) received normal drinking water (Figure 1(a)). Bottles were weighed daily. All procedures for handling animals were approved by the Animal Experimentation Committee of Gifu Women's University Graduate School of Human Life Science. PYC was provided by DKSH Japan K.K., Tokyo, Japan. CORT (4-pregnen-11*β* 21-DIOL-3 20-DIONE 21-hemisuccinate) was purchased from Sigma-Aldrich (Cat. number C6766, St. Louis, MO, USA), dissolved in tap water, and neutralized to a pH of 7.4.

### 2.2. FST

FST was performed with an acrylic cylinder (160 mm diameter, 225 mm height) filled to the height of 150 mm with water maintained at 24°C. Mice were placed in the cylinder for 6 min. Behavior was recorded with a video camera to measure immobility time when the mouse remained floating passively in the water (dog paddling in the water without struggling and only making movements necessary for keeping the head above the water) during the last 5 min, with the first 1 minute serving as a habituation. Immobility time (passive behavior) was interpreted as a measure of depressive-like behavior. The mice were then removed from the cylinder, dried with tissue paper, and returned to their home cage.

### 2.3. Statistics

Results are expressed as the mean ± SEM. All data were analyzed statistically using one-way analysis of variance (ANOVA), followed by a post hoc Dunnett's test. Statistical significance was set at a value of *P* < 0.05.

## 3. Results and Discussion

*Body Weights and Food Intake.* Body weights and food intake did not differ among the groups throughout the experimental period (Figures 1(b) and 1(c)). These results were the same as those of Gourley et al. [8]. It is considered that CORT-induced depression behavior is from mild stress. These results indicate that the CORT and PYC did not alter the body composition, and the influence of buoyancy and gravitational force in the water was ignored in the FST. Therefore, the results in our FST were comparable among 3 groups as depressive-like behavior.

**Figure 2:**
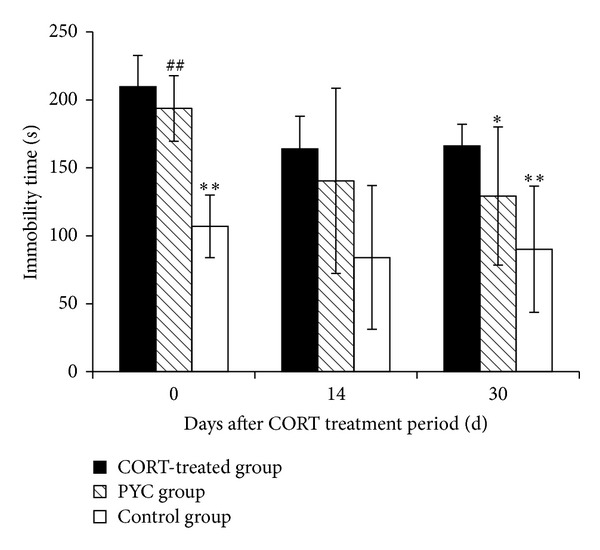
Effects of PYC on depression-like behavior. Results are presented as the mean ± SEM of six experiments: **P < 0.01 compared with CORT-treated group; *P < 0.05 compared with CORT-treated group; ^##^ *P* < 0.01 compared with control group.

*Effects of CORT and PYC on Depression-Like Behavior.* The effect of CORT on depression-like behavior was investigated by the FST. Immobility times in the PYC and CORT-treated groups were longer than in the control mice after the CORT treatment period, by post hoc analyses at day 0 (Figure 2). These times were about 200 sec and were the same as in a previous report [8]. Gourley et al. revealed that CORT increased immobility by selectively reduced pERK1/2 in the dentate gyrus and that the increased immobility time by CORT is not due to locomotor differences [8]. These results suggest that oral CORT exposure produced mild depression-like behavior in this study.

Immobility time of the PYC group was shortened 30 days after the CORT treatment period, but a difference did not appear after 14 days by one-way ANOVA (Figure 2). There is a report that it takes four weeks for the oxidative stress to be significantly lowered by PYC [9]. Post hoc analyses indicated that a significant difference was not recognized between PYC and the control group after 30 days (Figure 2). These results suggest that the depression-like behavior was improved by administration of PYC, almost to the control level. Treatment of glucocorticoid increases ROS in the brain of animals [10]. An excess level of ROS can damage cellular components and induce functional abnormalities in many cell types [11]. PYC scavenges free radicals and promotes cellular health [12] and increases the level of antioxidant enzymes [13]. Our results show that PYC supplements may reduce depression-like behavior in CORT mice model by its antioxidant activity.

In the future, we will examine antioxidant enzyme activities of PYC in depression-like behavior and confirm the effects of PYC with behavioral tests and their antioxidant effects.

## 4. Conclusions 

The present results suggest that chronic treatment of CORT induced mild stress-mediated depression-like behaviors as observed by FST and that the potent antioxidant activity of PYC can slightly decrease the progression of stress.
